# Association of plasma concentration of trace metals with frontotemporal degeneration

**DOI:** 10.3389/fneur.2025.1593821

**Published:** 2025-05-09

**Authors:** Kelly DeLano, Alex C. Sprague, Roman Jandarov, Brian P. Jackson, Rhonna Shatz, Scott M. Langevin, Russell P. Sawyer

**Affiliations:** ^1^Department of Neurology and Rehabilitation Medicine, University of Cincinnati College of Medicine, Cincinnati, OH, United States; ^2^Department of Environmental and Public Health Sciences, University of Cincinnati College of Medicine, Department of Environmental and Public Health Sciences, Division of Epidemiology, Cincinnati, OH, United States; ^3^Department of Environmental and Public Health Sciences, Division of Epidemiology, Cincinnati, OH, United States; ^4^Department of Earth Sciences, Dartmouth College, Hanover, NH, United States; ^5^Division of Hematology & Oncology, University of Vermont Larner College of Medicine, Burlington, VT, United States; ^6^University of Vermont Cancer Center, Burlington, VT, United States

**Keywords:** FTD, dementia, neurodegenerative, manganese, chromium, inductively-coupled plasma mass-spectrometry

## Abstract

**Objective:**

Compare the burden of heavy metals in plasma from people with frontotemporal degeneration (FTD) and healthy controls.

**Methods:**

A cross-sectional study of 14 FTD cases and 28 healthy controls recruited from the University of Cincinnati. Plasma samples were sent to the Trace Element Analysis Core at Dartmouth College for assessment of 24 metals or metalloids via inductively coupled plasma mass spectrometry (ICP-MS). Unconditional logistic regression models were performed with adjustments for age (centered at the median) and sex.

**Results:**

After adjusting for age and sex, there was a significant positive association of FTD with the highest tertile of Manganese (ORadjusted = 11.1, 95% CI: 1.57–132) and Chromium (ORadjusted = 9.86, 95% CI: 1.24–218). There was significant inverse associations observed between FTD and the highest tertile of Barium (ORadjusted = 0.06, 95% CI: <0.01–0.47) and Mercury (ORadjusted = 0.13, 95% CI: 0.01–0.74), with a significant inverse trend (ptrend = 0.03).

**Conclusion:**

Significant associations between plasma concentration of several trace metals and FTD. The significantly elevated levels of Manganese and Chromium may suggest a role of environmental exposure in the pathogenesis of FTD. However, larger, well-designed prospective studies, along with complementary experimental work, are needed to better elucidate this relationship.

## Introduction

Frontotemporal dementia (FTD) is a group of progressive neurodegenerative diseases presenting with changes in behavior, language, executive control, and motor symptoms (1). FTD is the most common neurodegenerative disease in people under 60 years of age (1). Approximately 60% of FTD patients are 45–64 years old at the time of onset (1, 2). While 33–50% of FTD cases with a family history have an autosomal dominant pattern of FTD, the majority of FTD cases are sporadic (3). The etiology for these sporadic cases remains unknown, though there is speculation that environmental risk factors could play a significant role.

Trace metals entail a heterogenous group of metals and metalloids (4), characterized by a high nuclear density with potential toxicity even at relatively low parts per billion (ppb) levels (5). Exposure to metals has been established as a risk factor for amyotrophic lateral sclerosis (ALS), a disease on the same pathologic spectrum as FTD [6; 7]. Additionally, trace metals have been shown to interact with the proteins TAR DNA-binding protein (*TDP-43*) and fused in sarcoma (*FUS*), two proteins with mutations associated with autosomal dominant FTD (6, 7). Significant research has been conducted on the relationship between trace metals and several types of neurodegenerative diseases including Alzheimer’s disease (AD) (8) and Parkinson’s disease (PD) (9). High levels of manganese exposure have shown to be linked to cognitive function and contribute to the pathogenesis of AD (8). Manganese toxicity has also shown to lead to parkinsonism including tremors, muscle spasms, tinnitus, and hearing loss (10). However, the relationship between trace metals and FTD remains unclear. There has been some limited evidence of a potential association between FTD and metals based on a recent epidemiologic study (11) that relied solely on self-reported occupational and dietary exposure. However, that study did not include a biological assessment of metals burden in the study subjects. As the first step to assessing a possible relationship between trace metals and FTD, we conducted a pilot study to cross-sectionally compare the burden of heavy metals in plasma from FTD cases relative to that of healthy controls.

## Materials and methods

### Standard protocol approvals, registrations, and patient consents

The study protocol was approved by the University of Cincinnati Institutional Review Board. Informed consent was obtained from all participants or was provided by a guardian/power-of-attorney for FTD participants when necessary. Enrollment was completed between September 2020 and August 2021.

### Participants

This was a cross-sectional pilot study of 14 FTD cases and 28 healthy controls. Participants with FTD were diagnosed with probable or definite FTD based on the Rascovsky criteria for behavioral variant FTD or Gorno-Tempini criteria for primary progressive aphasia (PPA) (12, 13). Participants were excluded if they had a concurrent neurodegenerative disorder (AD + FTD, Parkinson’s disease, or Lewy body disease) based on the diagnostic review of two behavioral neurologists at our center (RPS, RSS). Healthy controls were recruited from among friends and family of cases that did not have symptoms of cognitive impairment. Healthy controls were excluded if they or their informants reported cognitive decline in the previous year or if there was evidence from a screening visit suggesting a neurodegenerative disorder (including neuropsychological assessment or cerebrospinal fluid analysis with Alzheimer’s biomarkers) or clinically significant neurologic disorder as determined by the principal investigator. Healthy controls with a family history (3 degrees) of autosomal dominant neurodegenerative or neuropsychiatric diseases and individuals with a known mutation causing neurodegenerative disease were also excluded.

Cross-sectional whole blood was collected in EDTA tubes and centrifuged at 2,000x*g* at 4°C for 15 min to isolate plasma, which was immediately placed in storage at −80°C until further processing. Participants with FTD underwent commercial genetic testing through PreventionGenetics, LLC or Variantyx Inc. to evaluate the presence of pathogenic variants associated with FTD, including *C9orf72* repeat expansions (14–16), and *progranulin* (*GRN*) (17, 18) or *microtubule-associated protein tau* (*MAPT*) mutations (19, 20). This test was used to categorize but not exclude FTD participants.

### Measurement of metal and metalloid concentrations

Plasma samples were sent to the Trace Element Analysis Core at Dartmouth College for assessment of 24 metals or metalloids via inductively coupled plasma mass spectrometry (ICP-MS). Case and control samples were randomly ordered prior to ICP-MS. Measured elements included beryllium (Be), magnesium (Mg), aluminum (Al), vanadium (V), chromium (Cr), manganese (Mn), iron (Fe), nickel (Ni), copper (Cu), zinc (Zn), cobalt (Co), arsenic (As), selenium (Se), strontium (Sr), molybdenum (Mo), silver (Ag), cadmium (Cd), tin (Sn), antimony (Sb), barium (Ba), mercury (Hg), thallium (Tl), lead (Pb), and uranium (U).

Plasma samples were diluted 10X with 0.2% HNO_3_ prior to analysis by triple quadrupole ICP-MS (QQQ-ICP-MS, Agilent 8,900, Wilmington, DE). The ICP-MS operated in collision mode using helium (He) and reaction mode using oxygen and mass shift. All analytes were analyzed in He mode and reaction mode using oxygen. Concentrations for all analytes except Be, V and Cd were reported in He mode. Blank dilutions were performed during sample preparation and used to establish method detection limits based on 3X standard deviation of the blank concentrations. Trace Elements Serum L-2 serum reference (Seronorm, *Billingstad, NOR*) was used as matrix matched reference material and analyzed after every 10 samples during the run (*n* = 6). For Be, V, Ag and Tl, recoveries exceeded the acceptance limit (> 125% recovery). The average recovery for the other analytes was 101 ± 4%, minimum 81% (Mo) and maximum 125% (Cr).

### Statistical analysis

Comparison of study participant characteristics was completed using Mann–Whitney U test for age and Fisher’s Exact tests for sex and race. Univariate differences in mean levels of trace metals in plasma were compared between cases and controls using the Wilcoxon rank-sum test. Associations between metals and FTD were further assessed via unconditional logistic regression. For these analyses, the measured plasma level of each metal was categorized based on the respective tertiles among control participants. Unconditional logistic regression models were performed with adjustments for age (centered at the median) and sex. No corrections were made for multiple comparisons due to the limited sample size and hypothesis-generating nature of this study. All statistical analyses were performed in R version 4.3.0 using R Studio version 22.0.3.

## Results

A description of demographic and clinical characteristics for FTD cases (*n* = 14) and healthy control subjects (*n* = 28) is presented in Table 1. There were no significant differences in age or sex between cases and controls. However, there was a significant difference by race (*p* = 0.04), with 28.6% of controls identifying as Black, whereas none of the FTD patients were Black. Only one of the FTD cases was shown to be harbor an autosomal dominant mutation (7.1%).

**Table 1:** Demographics and clinical characteristics of study subjects.

Characteristic	FTD Cases	Controls	*p*-value
Median Age (IQR)	67.5 (67–70)	65.0 (57.5–72)	0.24^a^
Female, *n* (%)	3 (21.4%)	13 (46.4%)	0.18^b^
Non-Hispanic White, *n* (%)	14 (100.0%)	20 (71.4%)	0.04^b^
Current Smoker, *n* (%)	0 (0.0%)	1 (3.7%)	0.37^b^
Former Smoker, *n* (%)	4 (28.6%)	3 (11.1%)	
Never a Smoker, *n* (%)	10 (71.4%)	23 (85.2%)	
Autosomal dominant mutation^c^, *n* (%)	1 (7.1%)	-	

a Mann–Whitney U test,

b Fisher’s Exact test.

c Germline C9orf72 repeat expansion, GRN or MAPT mutation.

FTD, frontotemporal dementia; IQR, interquartile range.

**Figure 1:**
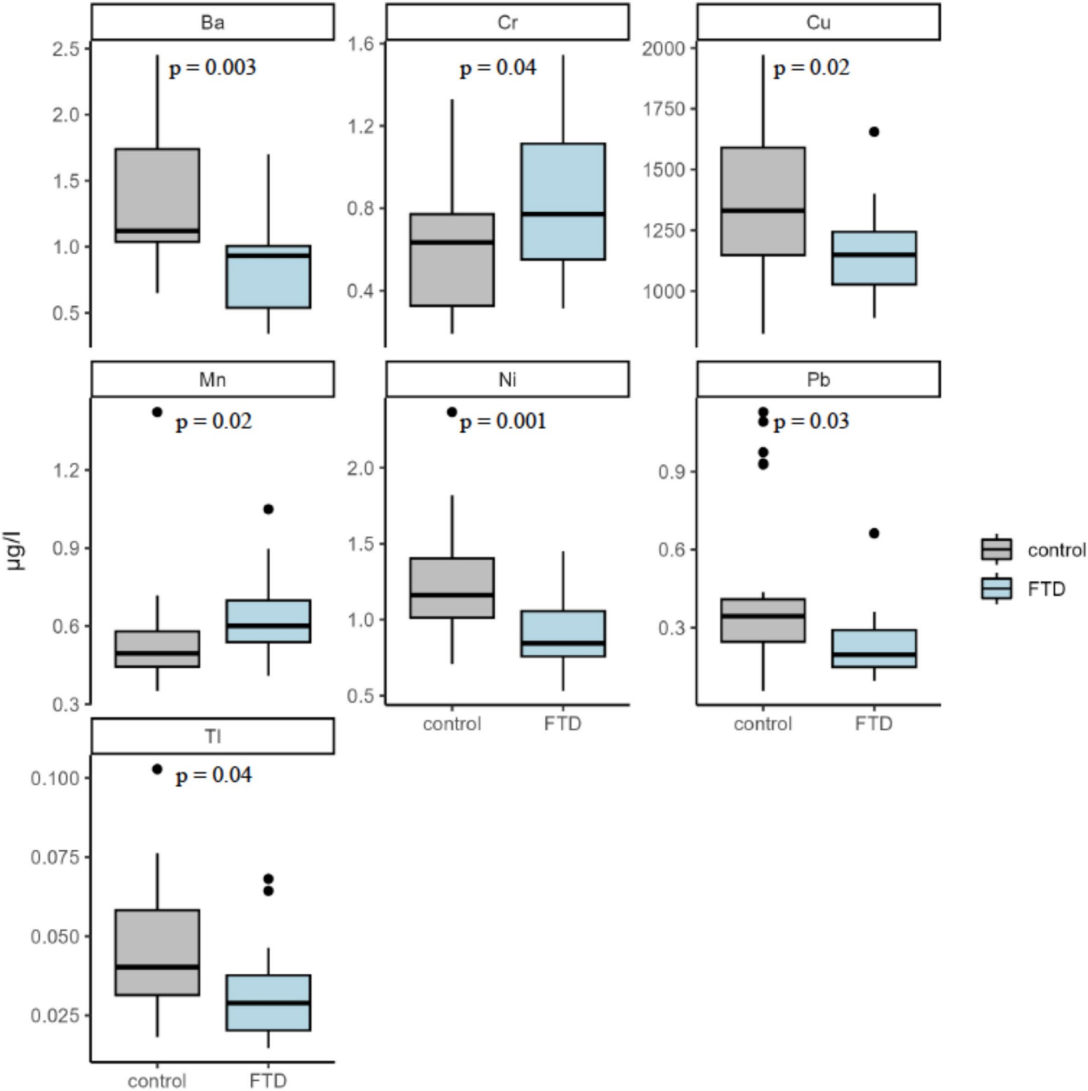
Plasma concentration of significantly different trace metals. Five metals were found to be significantly different between participants with FTD and controls: Barium (Ba), Chromium (Cr), Copper (Cu), Manganese (Mn), Nickel (Ni), Lead (Pb), and Thallium (Tl). Significance assessed by the Wilcoxon Rank-Sum test.

The median plasma concentration of 21 metals for FTD cases and controls are presented in Table 2. Be, Al, and U were omitted from the analysis due to measured concentration values for all samples falling below the respective lower detection limits of each assay. FTD cases had significantly higher median plasma concentrations of Cr (median: 0.771 μg/L for FTD cases vs. 0.635 μg/L for controls; *p* = 0.04) and Mn (0.601 μg/L for FTD cases vs. 0.496 μg/L for controls; *p* = 0.02). In contrast, FTD cases had significantly lower plasma concentrations of several metals compared with controls, including Ni (0.844 μg/L vs. 1.162 μg/L; *p* = 0.001), Cu (1,149.178 μg/L vs. 1,329.978 μg/L, *p* = 0.02), Ba (0.931 μg/L vs. 1.119 μg/L; *p* = 0.003), and Tl (0.029 μg/L vs. 0.040 μg/L; *p* = 0.04). Box plots comparing the distribution of these metals in plasma between cases and controls are presented in Figure 1.

**Table 2:** Median concentration (μg/L) of trace metals in plasma from frontotemporal dementia (FTD) cases and healthy controls.

Heavy metal	Cases	Controls	p_difference_^a^
Antimony (Sb)	6.377	4.747	0.10
Arsenic (As)	0.427	0.264	0.89
Barium (Ba)	0.931	1.119	0.003
Cadmium (Cd)	0.016	0.013	0.47
Chromium (Cr)	0.771	0.635	0.04
Cobalt (Co)	0.366	0.278	0.44
Copper (Cu)	1149.178	1329.978	0.02
Iron (Fe)	1425.099	1690.684	0.08
Lead (Pb)	0.195	0.344	0.03
Magnesium (Mg)	24095.139	25679.246	0.39
Manganese (Mn)	0.601	0.496	0.02
Mercury (Hg)	0.247	0.382	0.11
Molybdenum (Mo)	1.359	1.136	0.22
Nickel (Ni)	0.844	1.162	0.001
Selenium (Se)	154.708	163.573	0.31
Silver (Ag)	0.072	0.216	0.19
Strontium (Sr)	25.992	33.91	0.07
Thallium (Tl)	0.029	0.04	0.04
Tin (Sn)	0.201	0.194	0.72
Vanadium (V)	0.015	0.001	0.12
Zinc (Zn)	917.895	901.021	0.74

a Wilcoxon Rank-Sum test.

Unconditional logistic regression was employed to further model the association of FTD with the plasma level of each respective metal, with each metal concentration categorized according to the respective tertile in control subjects (Table 3). After adjusting for age and sex, there was a significant positive association of FTD with the highest levels of Mn (OR_adjusted_ = 11.1, 95% CI: 1.57–132) with a significant positive trend across increasing plasma levels (p_trend_ = 0.02); and the middle (OR_adjusted_ = 4.46, 95% CI: 3.96–86.1) and highest categories of Cr (OR_adjusted_ = 9.86, 95% CI: 1.24–218), again with a significant trend across levels (p_trend_ = 0.05). Conversely, there were significant inverse associations observed between FTD and the highest levels of Ba (OR_adjusted_ = 0.06, 95% CI: <0.01–0.47), with a significant inverse trend across increasing levels (p_trend_ = 0.01); Hg (OR_adjusted_ = 0.13, 95% CI: 0.01–0.74), with a significant inverse trend (p_trend_ = 0.03); and Pb (OR_adjusted_ = 0.12, 95% CI: 0.01–0.91), albeit the test for trend did not reach significance (p_trend_ = 0.06).

**Table 3:** Association of frontotemporal dementia with plasma concentration of trace metals.

Heavy metal	Tertile (μg/ml)	Crude OR (95% CI)	Adjusted OR (95% CI)^a^	p_trend_
Antimony (Sb)	Low	1.00 (reference)	1.00 (reference)	0.74
	Moderate	0.20 (0.01–1.58)	0.23 (0.01–1.96)	
	High	1.44 (0.35–6.33)	1.23 (0.27–5.72)	
Arsenic (As)	Low	1.00 (reference)	1.00 (reference)	0.93
	Moderate	0.50 (0.06–3.04)	0.58 (0.06–4.15)	
	High	1.40 (0.33–6.20)	0.98 (0.19–4.91)	
Barium (Ba)	Low	1.00 (reference)	1.00 (reference)	0.01
	Moderate	0.23 (0.03–1.17)	0.17 (0.02–1.02)	
	High	0.10 (<0.01–0.60)	0.06 (<0.01–0.47)	
Cadmium (Cd)	Low	1.00 (reference)	1.00 (reference)	0.31
	Moderate	0.68 (0.11–3.89)	0.71 (0.11–4.46)	
	High	1.75 (0.38–8.75)	2.38 (0.47–14.21)	
Chromium (Cr)	Low	1.00 (reference)	1.00 (reference)	0.05
	Moderate	4.50 (0.58–95.1)	4.46 (3.96–86.1)	
	High	8.00 (1.12–164.8)	9.86 (1.24–217.7)	
Cobalt (Co)	Low	1.00 (reference)	1.00 (reference)	0.52
	Moderate	0.22 (0.01–1.74)	0.21 (0.01–1.75)	
	High	1.78 (0.43–7.86)	1.63 (0.35–8.01)	
Copper (Cu)	Low	1.00 (reference)	1.00 (reference)	0.10
	Moderate	0.40 (0.08–1.70)	0.40 (0.08–1.81)	
	High	0.11 (0.01–0.77)	0.16 (0.01–1.53)	
Iron (Fe)	Low	1.00 (reference)	1.00 (reference)	0.08
	Moderate	0.42 (0.07–1.96)	0.53 (0.08–2.88)	
	High	0.22 (0.03–1.14)	0.19 (0.02–1.08)	
Lead (Pb)	Low	1.00 (reference)	1.00 (reference)	0.06
	Moderate	0.49 (0.10–2.11)	0.59 (0.11–2.92)	
	High	0.12 (0.01–0.85)	0.12 (0.01–0.91)	
Magnesium (Mg)	Low	1.00 (reference)	1.00 (reference)	0.31
	Moderate	0.63 (0.13–2.86)	0.58 (0.11–2.83)	
	High	0.48 (0.08–2.30)	0.42 (0.07–2.23)	
Manganese (Mn)	Low	1.00 (reference)	1.00 (reference)	0.02
	Moderate	1.67 (0.23–15.0)	3.48 (0.38–42.9)	
	High	5.00 (0.96–38.9)	11.1 (1.57–131.9)	
Mercury (Hg)	Low	1.00 (reference)	1.00 (reference)	0.03
	Moderate	0.20 (0.03–1.04)	0.21 (0.02–1.26)	
	High	0.18 (0.02–0.92)	0.13 (0.01–0.74)	
Molybdenum (Mo)	Low	1.00 (reference)	1.00 (reference)	0.66
	Moderate	1.56 (<0.01–2.45)	<0.01 (<0.01 - > 1,000)	
	High	1.80 (0.44–7.94)	1.46 (0.30–7.23)	
Nickel (Ni)	Low	1.00 (reference)	1.00 (reference)	0.11
	Moderate	0.18 (0.02–0.92)	0.21 (0.03–1.10)	
	High	0.20 (0.03–1.04)	0.29 (0.03–1.76)	
Selenium (Se)	Low	1.00 (reference)	1.00 (reference)	0.78
	Moderate	0.93 (0.20–4.15)	0.76 (0.15–3.60)	
	High	0.56 (0.09–2.79)	0.81 (0.13–4.83)	
Silver (Ag)	Low	1.00 (reference)	1.00 (reference)	0.31
	Moderate	0.42 (0.07–1.96)	0.56 (0.09–2.96)	
	High	0.42 (0.07–1.96)	0.44 (0.07–2.22)	
Strontium (Sr)	Low	1.00 (reference)	1.00 (reference)	0.46
	Moderate	0.28 (0.04–1.48)	0.33 (0.04–1.93)	
	High	0.56 (0.11–2.43)	0.60 (0.12–2.81)	
Thallium (Tl)	Low	1.00 (reference)	1.00 (reference)	0.17
	Moderate	0.45 (0.09–1.95)	0.57 (0.11–2.73)	
	High	0.25 (0.03–1.35)	0.28 (0.03–1.64)	
Tin (Sn)	Low	1.00 (reference)	1.00 (reference)	0.60
	Moderate	1.00 (0.21–4.81)	1.30 (0.25–6.97)	
	High	0.72 (0.14–3.56)	0.58 (0.09–3.40)	
Zinc (Zn)	Low	1.00 (reference)	1.00 (reference)	0.52
	Moderate	0.67 (0.11–3.54)	0.65 (0.10–3.80)	
	High	1.33 (0.30–6.15)	1.71 (0.34–9.57)	
Vanadium (V)	Low	1.00 (reference)	1.00 (reference)	0.22
	Moderate	0.43 (0.02–3.55)	0.51 (0.02–4.71)	
	High	2.31 (0.58–9.96)	2.46 (0.58–11.4)	

a Adjusted for age and sex.

OR = odds ratio; 95% CI = 95% confidence interval.

## Discussion

We observed significant associations between plasma concentration of several trace metals and FTD. Of note were the significantly elevated levels of Mn and Cr, which may suggest a role of environmental exposure in the pathogenesis of FTD. However, larger, well-designed prospective studies, along with complementary experimental work, are needed to better elucidate this relationship. To our knowledge, the first study to report differential concentrations of trace metals in plasma from FTD cases relative to that of healthy control subjects.

These findings are in-line with the existing literature reporting on associations of metals or metalloids with other forms of neurodegenerative diseases, including AD (8), PD (9) and the pathologically related ALS (21). Our findings are also consistent with the results from an epidemiologic study of 19 FTD cases and 54 healthy controls in which the authors reported elevated (albeit non-significant) associations between self-reported environmental and occupational exposures with FTD, including for occupational exposure to aluminum (11).

Manganese (Mn) is an essential ubiquitous trace element required for normal growth, development and cellular homeostasis; however, it can be toxic to the central nervous system in excessive levels. The routes of Mn exposure are mainly through dietary intake, dermal absorption, and inhalation (22). Overexposure from environmental sources can result clinical symptoms mimicking Parkinson’s disease (22). Additionally, there is increasing evidence has shown that Mn is potentially involved in the progression of Alzheimer’s disease, with Alzheimer’s disease patients having deregulated metabolism of Mn, and a dysfunction of the Mn-SOD scavenger system, associated with the formation of senile plaques (23). Furthermore, previous studies have shown that toenail concentrations of manganese correlated with difficulties in attention (24). One proposed mechanism astrocyte toxicity, astrocytes have a greater tendency to accumulate Mn than neurons and altered glial function and secondary impairment of astrocyte-dependent neuronal functions are observed in neurodegenerative disease (25).

Chromium (Cr) is the seventh most abundant element on earth and naturally occurs in two valence states, with the hexavalent state widely recognized as a human carcinogen and an environmental pollutant (26). Widespread neurodegeneration has been observed in multiple studies across multiple species, with notable toxicity to Purkinje cells of the cerebellum (27, 30, 31). Hegazy et al. observed a dose- and time-dependent significant decrease in the number of viable neurons in cortical gray matter, with increased reactive astrogliosis (27, 28). Particularly relevant to FTD is that some studies have shown higher Cr levels in the temporal lobes (26).

While we did not find an association between Cadmium and FTD, it is worth noting that cadmium is a highly toxic pollutant that permeates environmental, industrial, and agricultural spaces (8). Cadmium exposure has also been associated with neurodegenerative disease pathologies observed in Alzheimer’s disease (AD), Parkinson’s disease (PD), and amyotrophic lateral sclerosis (ALS) (9–11). Once within the nervous system, cadmium disrupts mitochondrial respiration by decreasing ATP synthesis and increasing the production of reactive oxygen species (8). Cadmium also impairs normal neurotransmitter release asynchronicity, disrupting neurotransmitter signaling proteins (12). Cadmium furthermore impairs the blood–brain barrier^13^ and alters the regulation of glycogen metabolism (14). Together, these mechanisms represent multiple sites of biochemical perturbation that result in cumulative nervous system damage which can increase the risk for neurological and neurodegenerative disorders (8).

Strengths of our pilot study include access to FTD patients and control subjects for collection of blood samples and systematic measurement of 24 metals and metalloids using validated ICP-MS assays. However, there are also several limitations to our study. The relatively small sample size limited our statistical power to detect significant associations and adversely affects study precision. The small sample size was a result of pilot awards and time frame for recruitment. Our sample size was relatively small, in biostatistical analysis we sought to balance correction for our small sample size with informative statistical modeling. Given the small sample size and non-normatively distributed data we used non-parametric test techniques such as the Mann–Whitney U Test rather than the Student T test. We utilized unconditional logistic regression, with healthy control vs. FTD as the outcome, rather than simple linear regression (with heavy metal concentration as the outcome) because it was more representative of our hypothesis that heavy metal concentrations influence the development of FTD rather than FTD influencing the concentration of heavy metals in the blood. Additionally, though our FTD and healthy control groups were comparable in terms of age and sex, our opinion was that controlling for these covariates increased the strength of our findings. Regardless, our small sample size may have limited the number of true relationships that we observed. Notwithstanding, we still identified several significant associations, lending credence to our hypothesized role of environmental exposure to metals as a risk factor for FTD. We also note that our study did not correct for multiple comparisons, increasing the risk of Type I (false positive) errors, however this was deemed necessary due to the discovery-focused nature of this study. Our pilot study was also limited in its cross-sectional analysis, which does not establish temporality of exposure and outcome. Further, we cannot determine if the altered metals levels are due to exogenous exposure, alterations in homeostasis, as others have proposed. Indeed, we observed inverse associations between plasma concentration of trace metals that may hint at the latter. Lastly, we were limited by insufficient sensitivity of assays for three metals with all samples falling below the lower detection limit. This unfortunately included Al, which has been implicated as a potential risk factor for neurodegenerative diseases (29).

Our study presents measured evidence of a potential relationship between FTD and Mn and Cr. Further research is needed to confirm these findings and determine how these metals interact with the TDP-43 and the FUS proteins to further explain the putative role between metals or metalloids and FTD prevalence pathogenesis.

## Data Availability

The elemental analysis data was generated at the Trace Element Analysis Core Facility at Dartmouth College. The raw data and final results supporting the findings of this study will be made publicly available through the AD Data Initiative Repository (https://www.alzheimersdata.org/ad-workbench/data-repository).
